# Suicidal Ideation and Non-suicidal Self-injury in Early Adolescence: The Role of ADHD Symptoms, Affect, and Emotion Regulation

**DOI:** 10.62641/aep.v54i3.2131

**Published:** 2026-06-15

**Authors:** Jon García-Ormaza, Karmele Salaberria, Amador Priede, César González-Blanch, Paloma Canduela, Ennio Ammendola, Jeffrey V. Tabares, Alexander Muela

**Affiliations:** ^1^Biobizkaia Health Research Institute, 48903 Barakaldo, Bizkaia, Spain; ^2^Osakidetza Basque Health Service, Zamudio Hospital, 48170 Zamudio, Bizkaia, Spain; ^3^Medical School, University of the Basque Country (UPV/EHU), 48940 Leioa, Bizkaia, Spain; ^4^Department of Clinical and Health Psychology and Research Methodology, University of the Basque Country (UPV/EHU), 20018 San Sebastián, Gipuzkoa, Spain; ^5^Biogipuzkoa Health Research Institute, 20014 San Sebastián, Gipuzkoa, Spain; ^6^Valdecilla Biomedical Research Institute (IDIVAL), 39011 Santander, Cantabria, Spain; ^7^Mental Health Strategy and Planning Office, Servicio Cántabro de Salud, 39011 Santander, Cantabria, Spain; ^8^Mental Health Centre, Hospital General Sierrallana, 39300 Torrelavega, Cantabria, Spain; ^9^Mental Health Centre, University Hospital Marqués de Valdecilla, 39008 Santander, Cantabria, Spain; ^10^Department of Psychology, International University of La Rioja (UNIR), 26006 Logroño, La Rioja, Spain; ^11^Faculty of Health Sciences, Universidad Europea del Atlántico, 39011 Santander, Cantabria, Spain; ^12^Consejería de Educación, Formación Profesional y Universidades, 39003 Santander, Cantabria, Spain; ^13^Department of Psychiatry, Larner College of Medicine, University of Vermont, Burlington, VT 05405, USA

**Keywords:** ADHD, emotional dysregulation, suicidal ideation, non-suicidal self-injury, adolescence

## Abstract

**Background:**

Suicidal ideation and non-suicidal self-injury (NSSI) are highly prevalent during early adolescence and represent serious public health concerns. Emotional dysregulation has been identified as a transdiagnostic factor underlying various risk behaviors, particularly in youth with externalizing difficulties such as attention-deficit/hyperactivity disorder (ADHD). While prior research has examined these factors independently, few studies have explored their joint and differential contribution to suicide-related outcomes in early adolescence. This study aimed to examine the association of ADHD-related symptoms, positive and negative affect, and emotional dysregulation with suicidal ideation and NSSI, as well as to explore whether emotional dysregulation moderates the relationship between ADHD-related symptoms and these risk behaviors.

**Methods:**

A total of 1079 Spanish adolescents (M_age_ = 12.6 years, SD = 0.6) enrolled in the first year of compulsory secondary education participated in a cross-sectional study. Standardized self-report measures were used to assess ADHD-related symptoms (Strengths and Difficulties Questionnaire [SDQ]), affect (Positive and Negative Affect Schedule for Children and Adolescents [PANAS-N]), and difficulties in emotion regulation (Difficulties in Emotion Regulation Scale [DERS-18]), alongside indicators of suicidal ideation and NSSI. Data analysis included group comparisons and hierarchical logistic regression models.

**Results:**

Suicidal ideation was reported by 10.1% of participants, and NSSI by 16.4%. Adolescents with either behavior exhibited significantly more hyperactivity-inattention symptoms, greater negative affect, lower positive affect, and substantially elevated emotional dysregulation. In regression analyses, ADHD-related symptoms remained significant predictors after accounting for affect, although emotional dysregulation emerged as the strongest predictor, reducing the effects of other variables. Specific dimensions such as lack of emotional clarity and non-acceptance of emotional responses were associated with suicidal ideation, while impulse control difficulties and lack of adaptive strategies predicted NSSI. No moderation effects were found.

**Conclusions:**

ADHD-related symptoms and emotional dysregulation both contribute independently to adolescent suicidal ideation and NSSI, with emotional dysregulation showing the most robust predictive value. School-based prevention efforts should incorporate emotion regulation skill-building to reduce suicide risk and NSSI in early adolescence.

## Introduction

Suicide in adolescence is a major public health concern worldwide, and it is one of the leading causes of death among teens and young adults [1]. Research has identified a positive association of impulsivity, hyperactivity, and attention deficits with suicidal behavior in both males and females and across different age groups [2]. Correlational studies [3] and various systematic reviews [2, 4, 5, 6] provide additional support for this relationship in adolescence, with high rates of suicidal ideation and behavior being observed among individuals with attention-deficit/hyperactivity disorder (ADHD). In fact, more than half of adolescents with ADHD have reported suicidal thoughts, and approximately one in ten have a history of suicide attempts [2].

Importantly, it is estimated that two-thirds of ADHD cases have at least one comorbid psychiatric diagnosis, the most common being conduct disorder, substance use, and major depression [2, 7], and this may also increase the risk of suicide. Furthermore, it is suggested that impulsivity, one of the core symptoms of ADHD, is a volitional factor in the transition from suicidal ideation to suicidal behavior. According to the integrated motivational-volitional model of suicide [8], impulsivity facilitates the transition to action by reducing the capacity to inhibit suicidal ideation, in other words, it lowers the behavioral barriers to acting on these thoughts.

In addition to the role of impulsivity, other risk factors for suicidal ideation and behavior in adolescents with ADHD have been identified. These include the severity and persistence of ADHD symptoms, family history of ADHD or other mental health conditions, adverse childhood experiences, and difficulties with social functioning [4]. Some studies have also found that females with ADHD are more likely to exhibit suicidal ideation and behavior than are males with the disorder [4].

Non-suicidal self-injury (NSSI) in adolescence is another important risk factor for suicidal behavior, and it is considered the most significant predictor of suicide during this life stage [9, 10]. It is estimated that around half of those who die by suicide have previously engaged in NSSI [9]. Furthermore, adolescents who engage in non-suicidal self-injury show a two- to four-fold increased likelihood of suicidal ideation and suicide attempts, as evidenced by longitudinal meta-analytic findings [11].

The risk of self-injury increases considerably in the presence of concurrent psychological variables such as entrapment, shame, rumination, hopelessness, thwarted belongingness, impulsivity, and lack of social support [12]. The repetition of NSSI in young people has also been linked to borderline personality disorder, mood disorders, and alcohol misuse [13].

The motivation for NSSI behavior is diverse and includes functions such as emotional relief, affect regulation, self-expression, identity validation, self-soothing, self-protection, and communication of distress [14]. From this functional perspective, it has been suggested that the transition from suicidal ideation to a suicide attempt can often be explained by NSSI ceasing to be effective as an emotion regulation strategy [15].

In this context, several studies have reported a close relationship between ADHD-symptoms and NSSI [16, 17]. This highlights the need for systematic assessment of these behaviors in adolescents with ADHD-related symptoms, as well as the importance of developing prevention strategies that are tailored to the emotional and behavioral characteristics of these young people.

### The Present Study

Despite the growing body of international evidence regarding the association between ADHD-related symptoms, suicidal ideation, and NSSI, few studies have examined this relationship in adolescents, especially those at the beginning of this life stage. It has been reported, for example, that a quarter of suicidal children under 12 years of age had ADHD, while around 10% of adolescents with ADHD have made at least one suicide attempt [2]. However, none of these studies have been conducted in the Spanish population. In the current context of growing concern about the mental health of adolescents in Spain, with reports of earlier hospitalization for mental health problems and an increased prevalence of both ADHD and suicidal behavior [18, 19, 20], this is an important gap in the literature.

It is also essential to identify the factors that may influence the relationship between ADHD-related symptoms and suicidal behavior so as to design more effective preventive interventions [21], especially within schools. In this respect, emotional dysregulation and emotional variability have been suggested as possible risk mechanisms in adolescents with ADHD-related symptoms [4, 22], who have greater difficulty managing their emotions, more frequently experience negative affect, and exhibit lower levels of positive affect in comparison with their peers without such symptoms [23, 24]. They are also prone to emotional lability/instability and impulsivity, characterized by rapid and intense fluctuations between positive and negative emotional states [25].

In light of the above, the specific study objectives were as follows:

1. To analyze the association of ADHD-related symptoms with suicidal ideation, controlling for the effect of gender, positive and negative affect, and emotional dysregulation.

2. To analyze the association of ADHD-related symptoms with NSSI, once again controlling for gender, positive and negative affect, and emotional dysregulation.

3. To explore whether emotional dysregulation moderates the relationship between ADHD-related symptoms and both suicidal ideation and NSSI.

The study hypotheses were as follows:

H1. Suicidal ideation will be more prevalent among adolescents with more ADHD-related symptoms, even after controlling for gender, positive and negative affect, and emotional dysregulation.

H2. The prevalence of NSSI will be higher among adolescents with more ADHD-related symptoms, even after controlling for gender, positive and negative affect, and emotional dysregulation.

H3. Emotional dysregulation will moderate the relationship between ADHD-related symptoms and both suicidal ideation and NSSI, such that the relationship will be stronger in adolescents with more difficulties in emotion regulation.

## Methods

### Participants

The initial sample comprised 1522 adolescents enrolled in the first year of compulsory secondary education across 24 schools in Cantabria, northern Spain. Due to missing data across study variables, the effective sample size varied across analyses. The maximum analytic sample included 1079 participants (M_a⁢g⁢e_ = 12.6 years, SD = 0.6; 48.2% female, 50.6% male, 0.9% non-binary, 0.3% gender not stated).

### Instruments

Self-Injurious Thoughts and Behaviors Interview-Revised (SITBI-R; [26]). The SITBI-R is a structured instrument that evaluates the presence, frequency, recency, and history of suicide and self-harming thoughts and behavior in adolescents and adults. It has been shown to be a reliable and valid tool for detecting these thoughts and behaviors in both clinical and community populations [26, 27]. For the present study, lifetime presence of suicide thoughts was assessed with the item “Have you ever had thoughts about killing yourself?”, with a yes/no response option. Those participants who answered yes to this question were then asked “When was the last time you had thoughts about killing yourself?”, with the response options being During the past month, During the past six months, During the past year, Over a year ago.

NSSI was assessed with the item “Have you ever had thoughts of purposely hurting yourself without wanting to die? (for example, cutting or burning)”, with a yes/no response option. Those participants who answered yes to this question were then asked “When was the last time you hurt yourself in this way?”, with the response options being During the past six months, During the past year, Over a year ago. Although the SITBI-R allows for a detailed assessment of multiple dimensions (e.g., presence, frequency, severity), in the present study suicidal ideation and NSSI were operationalized as binary indicators (presence vs. absence). This decision was guided by the population-based, school setting of the study and by the preventive focus on early detection of risk, where the presence of suicidal thoughts or self-injurious behaviors constitutes a clinically and educationally relevant threshold. The Spanish version of the SITBI has shown excellent inter-rater reliability for lifetime, past-year, and past-month presence/absence items (κ = 1.00) and excellent reliability for quantitative items (κ = 0.90), with good test–retest reliability for suicidal ideation (κ = 0.82), suicide plans (κ = 0.79), and suicide attempts (κ = 0.87), and moderate values for NSSI (κ = 0.65) and suicidal gestures (κ = 0.47) [28]. Evidence of construct validity has also been reported [28]. In addition, the questionnaire version of the SITBI has been used in recent studies with Spanish adolescent samples, supporting its applicability in this population [27]. Because outcomes were measured using single items, internal consistency indices are not applicable.

Difficulties in Emotion Regulation Scale (DERS-18; [29]). Difficulties in emotion regulation were assessed using the brief version of the DERS, which comprises 18 items distributed across six dimensions (three items each): Lack of emotional awareness, Lack of emotional clarity, Difficulties engaging in goal-directed behavior, Impulse control difficulties, Non-acceptance of emotional responses, and Limited access to emotion regulation strategies. Items are rated on a 5-point scale, from 1 (almost never) to 5 (almost always), with higher scores indicating greater difficulties in emotion regulation. The DERS-18 has shown excellent psychometric properties in clinical and non-clinical populations [29], and it has been used with adolescents with ADHD [23, 24]. The Spanish version of the DERS has been validated in adolescent populations. Gómez-Simón *et al*. [30] reported a six-factor structure consistent with the original model and demonstrated strict measurement invariance across sex in a large community sample of Spanish adolescents aged 12–18 years. Internal consistency for the subscales ranged from moderate to satisfactory (α = 0.71–0.88), with slightly lower reliability for the Awareness subscale (α = 0.62). In the present sample, internal consistency (Cronbach’s α) of scale scores was as follows: 0.77 for the Awareness and Clarity dimensions; 0.80 for Goals; 0.88 for Impulse; 0.83 for Non-acceptance; 0.82 for Strategies; and 0.91 for the total score.

Positive and Negative Affect Schedule for Children and Adolescents (PANAS-N; [31]). The PANAS-N (Spanish version of the PANAS for children and adolescents) evaluates positive and negative affect using a list of 20 emotion words, 10 relating to positive affect (e.g., interested, excited, attentive) and 10 to negative affect (e.g., distressed, nervous, irritable). Participants are asked to indicate how they usually feel or behave in relation to each emotion word, using a 3-point response scale ranging from 0 (Never) to 2 (Many times). The PANAS-N has shown a robust two-factor structure (positive and negative affect), good internal consistency (α = 0.72–0.75), and adequate convergent and discriminant validity with measures of anxiety and depression in community samples of Spanish adolescents [31]. In the present sample, internal consistency of scale scores was adequate for both positive affect (α = 0.77) and negative affect (α = 0.84).

Strengths and Difficulties Questionnaire (SDQ; [32]). The SDQ is a brief screening questionnaire for assessing emotional and behavioral difficulties in children and adolescents. It comprises 25 items distributed equally across five subscales that explore emotional difficulties, conduct problems, peer relationship problems, hyperactivity/inattention, and prosocial behavior. For the present study we used the Hyperactivity subscale, whose items evaluate behaviors such as inattention, restlessness, and impulsivity (e.g., “I am easily distracted, I find it hard to concentrate”). Items are scored on a 3-point scale (0 = Not true, 1 = Somewhat true, 2 = Certainly true), and hence the total subscale score ranges from 0 to 10, with a higher score indicating more hyperactivity symptoms or attention difficulties. The SDQ hyperactivity–inattention subscale is intended as a screening tool to assess ADHD-related symptomatology and does not constitute a clinical diagnosis of ADHD. The Spanish self-report version of the SDQ has demonstrated adequate psychometric properties in adolescent samples [33]. Confirmatory factor analyses support the five-factor structure of the instrument, and measurement invariance across gender and age has been established. Internal consistency for the SDQ subscales in Spanish adolescents has been reported as acceptable (α range = 0.71–0.75), with good reliability for the total difficulties score (α = 0.84) [33]. The SDQ is widely used in both clinical and community settings [33, 34], and it has also been applied in research on NSSI and suicide behavior in adolescence [17]. In the present sample, internal consistency of scores on the Hyperactivity subscale was acceptable (α = 0.75).

### Procedure

Schools were initially contacted by the Department of Education of the Government of Cantabria (Spain) to explain the objectives of the evaluation, request their collaboration, and obtain the necessary institutional authorization. For schools that agreed to participate, an information letter was subsequently sent to parents or legal guardians, describing the nature of the study and inviting their children to take part. Written informed consent was obtained from parents or legal guardians prior to data collection, and students also provided their assent. To ensure procedural consistency, the data collection protocol was standardized across all participating schools.

Questionnaire administration followed the same instructions, timing, and format in each school. All instruments were administered online during regular school hours, using a secure digital platform provided by the educational administration. Test administrators were authorized school or educational staff members who had received prior written instructions and guidance from the Department of Education regarding standardized administration procedures, confidentiality, and student support protocols. Questionnaires were completed in classroom settings under supervised conditions, with teachers or designated staff available to resolve procedural questions but instructed not to influence students’ responses. Recruitment took place between March and April 2025, and questionnaire administration occurred between May and June 2025.

Although previous research indicates that asking young people about suicidal thoughts does not increase the risk of suicidal behavior [26], specific measures were implemented to safeguard students’ emotional wellbeing during and after questionnaire completion. A psychologist or the school staff member responsible for students’ emotional welfare was available to respond to any distress and provide emotional support if needed. In cases where suicidal ideation with plan or intent, or recent suicidal behavior (within the past month), was identified, parents or legal guardians were immediately informed to ensure that an adult was aware of the situation and could take appropriate steps to guarantee the student’s safety. This safety protocol was communicated both verbally and in writing to students and their parents or legal guardians during the initial information session, prior to questionnaire administration.

Data management and quality control procedures were overseen by the Department of Education. Responses were collected electronically, anonymized prior to transfer, and stored on secure servers. The research team accessed only fully anonymized datasets and did not participate in data collection or data entry. Basic data integrity checks (e.g., missing data patterns, out-of-range values, duplicate cases) were conducted prior to analysis.

The study involved the retrospective analysis of anonymized data that had already been collected by the Department of Education of the Government of Cantabria for evaluative and preventive purposes. The study was approved by the Cantabria Medicine Research Ethics Committee (approval code: 2025.372) and was conducted in accordance with the ethical principles of the Declaration of Helsinki.

### Data Analysis

We began by performing a descriptive analysis of the main study variables, which included comparing mean scores according to the presence or absence of suicidal ideation and NSSI. Next, we conducted two binary logistic regression analyses with, respectively, suicidal ideation and NSSI as dependent variables (dichotomized: present/absent). ADHD-related symptoms, positive and negative affect, emotion regulation problems, and gender were included as predictor variables. To facilitate the interpretation of effects, we calculated and report β coefficients, standard error, odds ratios (OR) with their corresponding 95% confidence intervals, and *p* values. Regression models were specified following a theory-driven hierarchical approach grounded in prior research on ADHD-related symptoms, affective processes, and emotion regulation in adolescent suicidal behavior. In the first step, ADHD-related symptoms were entered to estimate their unadjusted association with suicidal ideation and NSSI. In subsequent steps, affective variables (positive and negative affect) and emotion regulation difficulties were added to examine whether these factors accounted for additional variance and attenuated the association between ADHD-related symptoms and the outcomes. Gender was included as a covariate in all models. The possible presence of multicollinearity between predictors was explored by calculating the variance inflation factor (VIF) and by analyzing model fit (Nagelkerke R^2^, Tjur R^2^, and the Akaike information criterion). Finally, and in order to examine a possible moderator effect, we tested an additional model which included the interaction between hyperactivity and emotional dysregulation. Regarding gender, although students could indicate male, female or non-binary on the questionnaire, the number who endorsed the latter option was too small to allow for valid statistical comparisons, and hence the analysis relates only to those students who identified as male or female. Missing data were examined prior to analysis. Across study variables, the proportion of missing values ranged from approximately 29% to 36%, with higher missingness observed for multi-item emotional measures. Given the school-based and voluntary nature of participation, this level of missingness was considered acceptable and consistent with previous large-scale adolescent surveys. All analyses were conducted in JASP (version 0.19.3; JASP Team, University of Amsterdam, Amsterdam, The Netherlands) using listwise deletion at the model level, such that each regression analysis included all cases with complete data for the variables involved in that specific model. No data imputation procedures were applied.

## Results

### Descriptive Analysis and Comparisons According to the Presence or Absence of Suicidal Ideation and NSSI

Lifetime suicidal ideation was reported by 10.1% of the early adolescents who responded to this question. Of these, 41.5% said they had thought about taking their own life during the past month, 25.5% during the past six months, 19.8% during the past year, and 13.2% over a year ago (see Table 1).

**Table 1.** **Baseline demographic and psychological characteristics of the sample**.

Variable		Total sample
N (maximum analytic sample)		1079
Age, years (M ± SD)		12.6 ± 0.6
Age range (years)		12–15
Grade		1st year of compulsory secondary education
Gender, n (%)		
	Female	520 (48.2%)
	Male	546 (50.6%)
	Non-binary	10 (0.9%)
	Not stated	3 (0.3%)
Suicidal ideation, n (%)		109 (10.1%)
NSSI, n (%)		176 (16.4%)
ADHD-related symptoms (SDQ-HI), M ± SD		4.37 ± 2.51
Emotion regulation difficulties (DERS), M ± SD		39.64 ± 14.16
Positive affect (PANAS-PA), M ± SD		13.06 ± 3.72
Negative affect (PANAS-NA), M ± SD		6.39 ± 4.33

Note: Percentages are based on participants who provided valid responses for each variable. No information on prior psychiatric diagnoses or mental health treatment was collected, as the study was conducted in a community-based school sample. ADHD, attention-deficit/hyperactivity disorder; NSSI, non-suicidal self-injury; PANAS-NA, negative affect; PANAS-PA, positive affect; SDQ-HI, hyperactivity–inattention.

Regarding gender, lifetime suicidal ideation was reported by 9.7% of girls (n = 50 of 515) and 10.3% of boys (n = 56 of 546) who answered this question, a difference that was not statistically significant (χ^2^ (1) = 0.094, *p* = 0.759). However, there were differences in the reported timeframe for thoughts of suicide: 50% of girls who reported suicidal ideation said they had had these thoughts within the past month, compared with 33.3% of the corresponding sub-group of boys. The majority of boys who reported suicidal ideation said this had occurred during the past six months (31.5%) or year (20.4%).

Table 2 shows comparisons of mean scores on the main study variables according to the presence or absence of suicidal ideation. It can be seen that there were significant differences on all the variables analyzed, with moderate or strong effect sizes.

**Table 2.** **Comparison of mean scores on the main study variables according to the presence or absence of suicidal ideation**.

Variable	Group not reporting ideation (n = 962)	Group reporting ideation (n = 108)	*t*	*df*	*p*	d
ADHD-related symptoms	4.14 (2.44)	6.13 (2.38)	–7.91	1046	< 0.001	0.82
Positive affect	13.28 (3.63)	11.31 (3.85)	5.03	998	< 0.001	0.54
Negative affect	5.88 (3.98)	10.95 (4.63)	–11.9	1004	< 0.001	1.25
Awareness	8.42 (3.24)	9.14 (2.92)	–2.19	1051	0.029	0.23
Clarity	5.84 (2.76)	9.61 (3.58)	–12.93	1052	< 0.001	1.32
Goals	7.51 (3.53)	11.02 (3.62)	–9.67	1040	< 0.001	0.99
Impulse	5.59 (3.11)	9.79 (3.86)	–12.89	1046	< 0.001	1.32
Non-acceptance	5.00 (2.69)	8.97 (3.67)	–13.68	1049	< 0.001	1.42
Strategies	5.31 (2.83)	9.60 (3.64)	–14.34	1046	< 0.001	1.47
DERS total	37.44 (12.36)	58.50 (14.97)	–15.69	962	< 0.001	1.67

Note: Values are reported as mean (standard deviation). Data are for the 1070 students who responded to this question. ADHD, attention-deficit/hyperactivity disorder; DERS, Difficulties in Emotion Regulation Scale.

Students who reported suicidal ideation scored significantly higher on ADHD-related symptoms (M = 6.13, SD = 2.38 vs. M = 4.14, SD = 2.44 in the group who had not had these thoughts), *t*(1046) = –7.91, *p* < 0.001, d = 0.82. Significant differences were also observed in relation to positive affect, and in this case, students who reported suicidal ideation scored lower (M = 11.31, SD = 3.85 vs. M = 13.28, SD = 3.63), *t*(998) = 5.03, *p* < 0.001, d = 0.54. Conversely, students who reported thoughts of suicide scored significantly higher on negative affect (M = 10.95, SD = 4.63 vs. M = 5.88, SD = 3.98), *t*(1004) = –11.90, *p* < 0.001, d = 1.25.

Regarding difficulties in emotion regulation, the group reporting suicidal ideation scored significantly higher on all subscales, especially those relating to non-acceptance of emotional responses, adaptive strategies, impulse control, and emotional clarity, where effect sizes were above 1.30. The total score for emotional dysregulation was also significantly higher among these students (M = 58.50, SD = 14.97 vs. M = 37.44, SD = 12.36), *t*(962) = –15.69, *p* < 0.001, d = 1.67, indicating a marked difference with respect to their peers who did not report having thoughts about suicide.

Lifetime NSSI was reported by 16.4% of the early adolescents who responded to this question. Of these, 56.8% said this had occurred during the past six months, 20.1% during the past year, and 23.1% over a year ago. Although NSSI was more common among boys (17.8%) than girls (14.5%), this difference was not statistically significant (χ^2^ (1) = 2.12, *p* = 0.146).

Group comparisons based on the presence or absence of NSSI revealed significant differences on all the variables considered (see Table 3). Students who reported NSSI scored significantly higher on ADHD-related symptoms and on negative affect, whereas their scores on positive affect were significantly lower. Regarding difficulties in emotion regulation, the NSSI group scored significantly higher on all subscales, especially those relating to impulse control, adaptive strategies, emotional clarity, and non-acceptance of emotional responses. The total score on the DERS-18 was also notably higher in this group (M = 52.79, SD = 15.98 vs. M = 37.13, SD = 12.31), with a very strong effect size (d = 1.21).

**Table 3.** **Comparison of mean scores on the main study variables according to the presence or absence of NSSI**.

Variable	Group not reporting NSSI (n = 899)	Group reporting NSSI (n = 176)	*t*	*df*	*p*	d
ADHD-related symptoms	4.08 (2.45)	5.79 (2.30)	–8.48	1060	< 0.001	0.7
Positive affect	13.34 (3.61)	11.58 (4.00)	5.57	1012	< 0.001	0.48
Negative affect	5.84 (3.97)	9.29 (4.99)	–9.76	1017	< 0.001	0.83
Awareness	8.41 (3.25)	8.98 (3.05)	–2.16	1053	0.031	0.18
Clarity	5.77 (2.72)	8.59 (3.68)	–11.61	1054	< 0.001	0.97
Goals	7.44 (3.52)	10.14 (3.77)	–9.01	1042	< 0.001	0.76
Impulse	5.47 (2.97)	8.88 (4.19)	–12.79	1048	< 0.001	1.07
Non-acceptance	4.99 (2.68)	7.50 (3.85)	–10.31	1051	< 0.001	0.87
Strategies	5.24 (2.80)	8.40 (3.78)	–12.69	1048	< 0.001	1.06
DERS total	37.13 (12.31)	52.79 (15.98)	–13.78	964	< 0.001	1.21

Note: Values are reported as mean (standard deviation). Data are for the 1075 students who responded to this question. ADHD, attention-deficit/hyperactivity disorder; DERS, Difficulties in Emotion Regulation Scale; NSSI, non-suicidal self-injury.

### Association of ADHD-related Symptoms and Emotional Variables With Suicidal Ideation

To identify emotional and behavioral factors associated with suicidal ideation in early adolescence, we estimated five binary logistic regression models to examine whether ADHD-related symptoms and emotional variables (affect and emotional dysregulation) predicted suicidal ideation, with gender being entered as a covariate (see Table 4). In all models, suicidal ideation was coded as a dichotomous variable (0 = No, 1 = Yes). Collinearity diagnostics indicated acceptable levels of multicollinearity in all fully adjusted models. VIF values ranged from 1.08 to 1.96 in models including the DERS total score, and from 1.23 to 2.93 in models including DERS subscales, indicating no evidence of problematic multicollinearity.

**Table 4.** **Results of the logistic regression analysis for suicidal ideation**.

Variable	Model 1 OR (*p*)	Model 2 OR (*p*)	Model 3 OR (*p*)	Model 4 OR (*p*)	Model 5 OR (*p*)
ADHD-related symptoms	1.39, 95% CI [1.27, 1.51] (< 0.001)	1.39, 95% CI [1.27, 1.51] (< 0.001)	1.18, 95% CI [1.06, 1.31] (0.002)	1.11, 95% CI [0.99, 1.25] (0.074)	1.15, 95% CI [1.01, 1.30] (0.029)
Gender (male)	—	1.01, 95% CI [0.66, 1.53] (0.967)	1.85, 95% CI [1.11, 3.09] (0.018)	1.70, 95% CI [0.97, 2.97] (0.065)	1.85, 95% CI [1.03, 3.34] (0.041)
Positive affect	—	—	0.92, 95% CI [0.86, 0.99] (0.017)	0.97, 95% CI [0.90, 1.04] (0.405)	0.96, 95% CI [0.89, 1.04] (0.297)
Negative affect	—	—	1.25, 95% CI [1.18, 1.33] (< 0.001)	1.08, 95% CI [1.00, 1.17] (0.057)	1.08, 95% CI [0.99, 1.17] (0.079)
DERS total	—	—	—	1.07, 95% CI [1.05, 1.10] (< 0.001)	—
Awareness	—	—	—	—	0.99, 95% CI [0.90, 1.10] (0.841)
Clarity	—	—	—	—	1.13, 95% CI [1.02, 1.25] (0.017)
Goals	—	—	—	—	1.02, 95% CI [0.92, 1.12] (0.764)
Impulse	—	—	—	—	1.04, 95% CI [0.94, 1.15] (0.460)
Non-acceptance	—	—	—	—	1.11, 95% CI [1.01, 1.23] (0.025)
Strategies	—	—	—	—	1.09, 95% CI [0.96, 1.23] (0.188)
Nagelkerke R^2^	0.115	0.115	0.253	0.321	0.333

ADHD, attention-deficit/hyperactivity disorder; DERS, Difficulties in Emotion Regulation Scale; OR, odds ratio.

In model 1, ADHD-related symptoms significantly predicted suicidal ideation (OR = 1.39, *p* = .0001) and explained 11.5% of the variance in this variable (Nagelkerke R^2^ = 0.115). This effect was maintained when introducing gender as a covariate in model 2, although the latter variable was not a significant predictor (*p* = 0.967).

In model 3, which included positive and negative affect, ADHD-related symptoms continued to be a significant predictor of suicidal ideation (*p* = 0.002), in addition to negative affect (OR = 1.25, *p* < 0.001) and gender (OR = 1.85, *p* = 0.018). Positive affect had a protective effect (OR = 0.92, *p* = 0.017). This model explained 25.3% of the variance in suicidal ideation (Nagelkerke R^2^ = 0.253).

The results for model 4 indicated that the total score on emotional dysregulation (DERS-18) was strongly associated with suicidal ideation (OR = 1.07, *p* < 0.001). In this model, the effect of ADHD symptoms decreased (*p* = 0.074), as did the effect of gender (*p* = 0.065) and negative affect (*p* = 0.057), which suggests that emotional dysregulation explains a sizable proportion of the effect observed in the previous models.

Finally, in model 5 we explored the role of different dimensions of emotional dysregulation. The results showed that a lack of emotional clarity (OR = 1.13, *p* = 0.017) and non-acceptance of emotional responses (OR = 1.11, *p* = 0.025) were significantly associated with suicidal ideation. ADHD-related symptoms continued to have a significant effect (OR = 1.15, *p* = 0.029), as did gender (OR = 1.85, *p* = 0.041). Neither positive nor negative affect reached statistical significance in this model. Other dimensions of emotional dysregulation, such as lack of impulse control and adaptive strategies, were not significantly associated with suicidal ideation when introduced simultaneously into the model.

Finally, we used interaction models to explore whether emotional dysregulation moderated the relationship between ADHD-related symptoms and suicidal ideation. Specifically, we tested logistic regression models with interaction terms between ADHD-related symptoms and the total DERS-18 score, as well as with the dimensions of emotional dysregulation that emerged as significant in the previous models (lack of emotional clarity and non-acceptance of emotional responses). In none of the models was the interaction term significant, which suggests that the effect of ADHD-related symptoms on suicidal ideation is additive and does not depend on the level of emotional dysregulation. Thus, although both ADHD-related symptoms and emotional dysregulation independently increase the risk of suicidal ideation, there appear to be no moderator effects between them.

### Association of ADHD-related Symptoms and Emotional Variables With NSSI

To examine whether NSSI in early adolescence was predicted by ADHD symptoms, affect (positive and negative), emotional dysregulation, and gender, we again tested five binary logistic regression models (see Table 5). Collinearity diagnostics indicated acceptable levels of multicollinearity in all fully adjusted models for both suicidal ideation and NSSI. VIF values ranged from 1.08 to 1.98 in models including the DERS total score, and from 1.21 to 2.93 in models including DERS subscales, indicating no evidence of problematic multicollinearity among predictors.

**Table 5.** **Results of the logistic regression analysis for NSSI**.

Variable	Model 1 OR (*p*)	Model 2 OR (*p*)	Model 3 OR (*p*)	Model 4 OR (*p*)	Model 5 OR (*p*)
ADHD-related symptoms	1.34, 95% CI [1.24, 1.43] (< 0.001)	1.33, 95% CI [1.24, 1.43] (< 0.001)	1.21, 95% CI [1.12, 1.32] (< 0.001)	1.18, 95% CI [1.08, 1.30] (< 0.001)	1.22, 95% CI [1.10, 1.34] (< 0.001)
Gender (male)	—	1.22, 95% CI [0.87, 1.72] (0.256)	1.86, 95% CI [1.24, 2.79] (0.003)	1.92, 95% CI [1.22, 3.01] (0.005)	1.94, 95% CI [1.21, 3.11] (0.006)
Positive affect	—	—	0.92, 95% CI [0.88, 0.97] (0.002)	0.98, 95% CI [0.92, 1.03] (0.421)	0.94, 95% CI [0.89, 1.00] (0.068)
Negative affect	—	—	1.17, 95% CI [1.12, 1.23] (< 0.001)	1.05, 95% CI [0.99, 1.12] (0.137)	1.04, 95% CI [0.98, 1.12] (0.204)
DERS total	—	—	—	1.06, 95% CI [1.04, 1.08] (< 0.001)	—
Awareness	—	—	—	—	0.92, 95% CI [0.85, 1.00] (0.054)
Clarity	—	—	—	—	1.09, 95% CI [1.00, 1.18] (0.055)
Goals	—	—	—	—	0.97, 95% CI [0.89, 1.05] (0.393)
Impulse	—	—	—	—	1.13, 95% CI [1.04, 1.22] (0.004)
Non-acceptance	—	—	—	—	1.05, 95% CI [0.96, 1.14] (0.284)
Strategies	—	—	—	—	1.11, 95% CI [1.00, 1.22] (0.050)
Nagelkerke R^2^	0.111	0.113	0.217	0.295	0.325

ADHD, attention-deficit/hyperactivity disorder; DERS, Difficulties in Emotion Regulation Scale; NSSI, non-suicidal self-injury; OR, odds ratio.

In model 1, ADHD-related symptoms were significantly associated with an increased likelihood of NSSI (OR = 1.34, *p* < 0.001), and explained 11.1% of the variance in this variable (Nagelkerke R^2^ = 0.111). This association was maintained when gender was introduced as a covariate (model 2), although the latter did not emerge as a significant predictor (*p* = 0.256).

In model 3, which included positive and negative affect, ADHD-related symptoms continued to be a significant predictor of NSSI (OR = 1.21, *p* < 0.001), along with gender (OR = 1.86, *p* = 0.003) and negative affect (OR = 1.17, *p* < 0.001). Positive affect showed a protective effect (OR = 0.92, *p* = 0.002). This model explained 21.7% of the variance in NSSI (Nagelkerke R^2^ = 0.217).

The results for model 4 indicated that the total score on emotional dysregulation (DERS-18) was significantly associated with NSSI (OR = 1.06, *p* < 0.001). In this model, ADHD-related symptoms (OR = 1.18, *p* = 0.0001) and gender (OR = 1.92, *p* = 0.005) remained significant, whereas the effects of negative affect (*p* = 0.137) and positive affect (*p* = 0.421) ceased to be statistically significant.

Finally, in model 5 the six dimensions of emotional dysregulation (DERS-18 subscales) were introduced as independent predictors. In this more complete model (Nagelkerke R^2^ = 0.325), ADHD-related symptoms (OR = 1.22, *p* < 0.001) and gender (OR = 1.94, *p* = 0.006) remained significant predictors of NSSI. Of the six difficulties in emotion regulation, poor impulse control (OR = 1.13, *p* = 0.004) and a lack of adaptive strategies (OR = 1.11, *p* = 0.050) emerged as significant predictors.

Finally, we explored whether emotional dysregulation moderated the relationship between ADHD-related symptoms and NSSI. The interaction between ADHD-related symptoms and the total DERS-18 score was not significant (*p* = 0.452), which suggests that the effect of ADHD-related symptoms on the presence of NSSI does not vary depending on the overall level of emotional dysregulation.

We then conducted additional analyses for the two sub-dimensions of emotional dysregulation that had been shown in previous models to have a significant direct effect: difficulty with impulse control and a lack of adaptive strategies. The results showed that while ADHD-related symptoms and both these sub-dimensions of emotional dysregulation were significantly associated with NSSI, the interactions between them were not statistically significant.

## Discussion

The primary aim of this study was to examine the relationship between ADHD-related symptoms, suicidal ideation, and NSSI in a sample of Spanish early adolescents, analyzing the role of emotional factors (affect and emotional dysregulation) and their possible moderating effect. The results provide solid evidence for both the high prevalence of these behaviors in early adolescence and the emotional mechanisms involved.

The descriptive results confirmed that suicidal ideation and NSSI are highly prevalent among adolescents in their first year of secondary education. Specifically, 10.1% of students reported having actively thought about suicide at least once during their life, while 16.4% had engaged in NSSI. These rates are in line with studies conducted in adolescent populations from other countries [9, 14, 35], and they highlight the urgent need to implement early preventive measures.

Overall, we observed no significant gender differences in the prevalence of suicidal ideation and NSSI, although there were some interesting variations in the time frame for the most recent experiences of this kind: whereas girls most commonly reported suicidal ideation within the past month, the most recent occurrence among boys was more likely to be at least six months previously. This pattern contrasts somewhat with much of the existing literature, which has consistently shown higher prevalence rates of suicidal ideation and NSSI among adolescent girls, often interpreted as reflecting greater internalization of emotional distress and a higher propensity to verbalize such experiences [36].

Several factors may help contextualize the absence of overall gender differences observed in the present study. First, the relatively young age of the sample may be particularly relevant, as gender differences in suicidal ideation and NSSI have been shown to become more pronounced during mid-to-late adolescence, while findings in early adolescence are often less consistent [36, 37]. Second, the cultural and educational context in which the data were collected may influence norms surrounding emotional expression and disclosure, potentially shaping how boys and girls report suicidal thoughts or NSSI behaviors [38]. Finally, the use of self-report measures administered in school settings may attenuate gender differences, either by facilitating disclosure among boys or by constraining more gender-differentiated patterns of distress [39]. Taken together, these considerations suggest that the present findings should be interpreted with caution and underscore the need for longitudinal research to examine how gender-related differences in suicidal ideation and NSSI may evolve across subsequent stages of adolescence.

Regarding our first study hypothesis, the results suggest that ADHD-related symptoms are associated with suicidal ideation in early adolescence, even after controlling for positive and negative affect and emotional dysregulation. This direct association, which was consistently observed in the initial regression models, is in line with previous studies that have reported increased vulnerability to suicidal spectrum behaviors among young people with ADHD symptoms [2, 4, 5, 6].

Notably, the progressive inclusion of the emotional variables in the regression models added further nuance to these results. When gender was examined within the multivariate models, an apparent divergence from the unadjusted findings became evident. Although no overall gender differences in suicidal ideation were observed in unadjusted gender comparisons—an outcome that is common in early adolescence—male gender emerged as a significant predictor once ADHD-related symptoms, affective variables, and emotional dysregulation were taken into account. This pattern suggests that emotional factors may amplify the risk of suicidal ideation in boys when they co-occur with ADHD-related symptoms. In other words, while gender differences may not be evident in unadjusted prevalence rates during early adolescence, the accumulation of emotional vulnerability appears to increase the likelihood of suicidal ideation among boys when these factors are jointly considered.

Within this multivariate context, affective variables played a central role in explaining suicidal ideation. Specifically, negative affect was associated with a greater likelihood of suicidal ideation, whereas positive affect had a protective effect. These findings support the hypothesis that emotional experience is a key risk factor for suicidal behavior in adolescents [27], insofar as a predominance of negative affect and the absence of positive emotions may trigger suicidal ideation, and vice-versa [40], while also limiting the young person’s capacity to regulate and cope with their mental pain. However, the most robust predictor in the more complex models was emotional dysregulation, with the total DERS-18 score explaining a considerable proportion of the variance that had previously been attributed to ADHD-related symptoms and negative affect. This result supports the hypothesis that difficulties in emotion regulation are a key factor in the emergence and persistence of suicidal ideation, with the latter, for some individuals, becoming a way of regulating their emotions [41].

When examining the specific dimensions of emotional dysregulation, lack of emotional clarity and non-acceptance of emotional responses emerged as the factors most closely associated with suicidal ideation. These difficulties could increase the risk of suicidal ideation and behaviors by making it hard for the young person to identify and understand their mental pain, to the extent that they end up rejecting their emotions and become trapped in a spiral of inner suffering. In fact, meta-analytic evidence indicates that individuals with greater difficulties in recognizing, understanding, and managing their emotions are approximately three times more likely to engage in NSSI than those with better emotion regulation skills [42]. Although other dimensions of emotional dysregulation such as impulsivity or a lack of adaptive strategies were associated with significant differences between adolescents who reported suicidal ideation and those who did not, these variables did not retain their predictive effect when entered jointly into the multivariate model. This suggests that the impact of these dimensions may overlap with that of other more central components of emotion regulation, such as clarity or acceptance of emotions.

Finally, the interaction models provided no evidence of a moderator effect of emotional dysregulation on the relationship between ADHD-related symptoms and suicidal ideation. This indicates that the two factors have an additive effect rather than interacting with one another. In other words, ADHD-related symptoms and emotional dysregulation independently heighten the risk of suicidal ideation, without mutually reinforcing their respective effects. This underscores the need for specific assessment of both these variables within educational settings, even if one or the other has already been identified as present.

The results of this study also reveal a significant association between ADHD-related symptoms and NSSI in early adolescence, in line with previous studies that have found a high prevalence of NSSI behaviors among young people with ADHD symptoms [17, 18]. This association remained robust even after introducing positive and negative affect and emotional dysregulation into the regression model, thereby underlining the independent contribution of externalizing symptoms to these behaviors.

As in the case of suicidal ideation, negative affect was observed to have a facilitator effect on NSSI, whereas positive affect acted as a protective factor. However, when emotional dysregulation was introduced into the model, the effect of both negative and positive affect decreased considerably and was no longer statistically significant. This suggests that difficulties in managing, accepting, and modulating emotions constitute a more proximal and powerful mechanism when it comes to explaining NSSI, a hypothesis that is consistent with functional models in which NSSI has been conceptualized as being associated with affect regulation processes [42].

The total DERS-18 score was positively and significantly associated with the likelihood of NSSI, and its inclusion in the regression model explained a sizable additional percentage of the variance. However, a more detailed analysis by sub-dimensions brought nuance to the findings, insofar as poor impulse control and a lack of adaptive emotion regulation strategies were the only dimensions to maintain a significant association with NSSI behaviors in the multivariate model, a finding that has been reported previously in systematic reviews [42]. This highlights the importance of helping adolescents to develop effective emotion regulation skills, especially in light of research showing that the perceived ineffectiveness of NSSI as an emotion regulation strategy may be a key factor in its maintenance and in the eventual transition to a suicide attempt [15, 43].

The absence of significant moderator effects between ADHD-related symptoms and emotional dysregulation once again suggests an additive effect of these factors, in other words, both ADHD-related symptoms and difficulties in emotion regulation contribute independently to the risk of NSSI. From a prevention perspective, these results underscore the need to address problems relating to behavioral control and inhibition, while also ensuring that emotionally vulnerable adolescents or those who exhibit externalizing symptoms are supported in developing more adaptive emotion regulation strategies [44].

From a developmental psychopathology perspective [45], the pattern of findings observed in this study should be interpreted in light of the specific characteristics of early adolescence. The transition from childhood to adolescence is characterized by dramatic shifts in social functioning and autonomy, accompanied by ongoing maturation of cortical and subcortical limbic regions involved in the processing of socio-affective information, which together increase the salience of socio-emotional stressors during this developmental period [46, 47]. At the functional level, early adolescence is also marked by continued maturation of executive control and emotion regulation capacities, alongside heightened emotional reactivity and increasing academic and social demands [48, 49]. As a result, some adolescents may experience a temporary imbalance in which emotion-driven processes develop more rapidly than top-down regulatory capacities [50].

Importantly, the specific emotion regulation dimensions associated with each outcome also align with a developmental interpretation. Difficulties related to emotional clarity and non-acceptance were more closely linked to suicidal ideation, which may reflect challenges in identifying, interpreting, and integrating internal emotional states during early adolescence—a developmental period characterized by heightened socio-affective salience and ongoing maturation of regulatory and integrative emotional processes [51]. By contrast, impulse control difficulties and limited access to adaptive regulation strategies were more strongly associated with NSSI. This pattern is also consistent with developmental neuroscience accounts of NSSI escalation in adolescence. For example, Cummings *et al*. [46] propose that heightened sensitivity to socio-affective pain may facilitate the onset of NSSI during adolescence, whereas increased sensitivity to reward—particularly to the relief obtained from escaping aversive socio-affective states—may strengthen the maintenance of NSSI through negative reinforcement learning. In this context, our finding that NSSI was more strongly associated with impulse-control difficulties and limited access to adaptive regulation strategies may reflect a tendency toward rapid, affect-driven responses that provide immediate relief when more deliberate regulatory strategies are not readily available.

Taken together, these findings suggest that early adolescence represents a critical window of vulnerability in which the co-occurrence of ADHD-related symptoms and still-developing emotion regulation capacities is associated with an increased likelihood of suicidal ideation and NSSI [52, 53]. However, given the cross-sectional design of the study, these associations should be interpreted as correlational and contemporaneous developmental co-occurrences rather than as directional or mechanistic processes. Longitudinal research will be necessary to determine the temporal ordering of ADHD-related symptoms, specific emotion regulation difficulties, and suicidal ideation or NSSI, as well as to examine how these associations evolve across subsequent stages of adolescence.

This study has several limitations that need to be considered when interpreting the results. First, the risk of suicidal ideation and NSSI was evaluated using self-report instruments. Although this approach provides valuable information from the perspective of adolescents themselves, the results may be affected by social desirability bias and are not directly comparable with those obtained through clinical interviews or other structured methods. A related limitation concerns the operationalization of suicidal ideation and NSSI as binary variables derived from SITBI-R items. While this approach facilitates feasibility and interpretability in large, school-based samples, it does not capture the full heterogeneity of these phenomena in terms of frequency, severity or chronicity. As a result, variability within each outcome category may have been underestimated, potentially attenuating the strength of the observed associations. Future studies should incorporate dimensional and longitudinal assessments of suicidal and self-injurious behaviors to better characterize developmental risk trajectories across adolescence. A further limitation concerns the relatively high proportion of missing data across some study variables, which ranged from approximately 29% to 36%, particularly for multi-item emotional measures. Because analyses were conducted using listwise deletion, this may have reduced statistical power and potentially affected the representativeness of the analytic sample. Although this approach is commonly used in large-scale, school-based surveys, it may introduce bias if data are not missing completely at random. Accordingly, the present findings should be interpreted with caution. Future research should apply more robust missing data techniques, such as multiple imputation, to better assess the stability of these associations and minimize potential bias related to missingness. It should also be noted that in the present study the SDQ was only completed by adolescents. For a more complete and accurate assessment of ADHD-related (hyperactivity–inattention) symptoms and emotion regulation difficulties, future research should include multi-informant data from parents and teachers. A further limitation concerns the cross-sectional design, which prevents us from establishing causal relationships between the variables analyzed. Although we identified significant associations between ADHD-related symptoms, the emotional variables, and suicidal ideation and NSSI, it is not possible to determine the directionality or temporality of these relationships. Additional studies using longitudinal designs are required to examine how these variables change over time and to clarify the causal relationships between them. Finally, although we tested interaction models to explore the possible moderating effect of emotional dysregulation on the relationship between ADHD symptoms and suicidal ideation or NSSI, the interaction terms were not significant. This suggests that ADHD-related symptoms and emotional dysregulation have additive effects, rather than interacting with one another. However, replicating these analyses in larger samples or with more advanced statistical techniques (e.g., structural equation modeling) might reveal more complex relationships between these variables.

## Conclusions

Overall, the findings of this study provide robust evidence for the association between ADHD-related symptoms, emotional difficulties, and suicidal ideation and non-suicidal self-injury in early adolescence. Adolescents presenting a combination of behavioral, cognitive, and emotional risk factors appear to constitute a particularly vulnerable group, with emotion dysregulation emerging as a key factor associated with both outcomes. Importantly, different dimensions of emotion dysregulation were differentially related to suicidal ideation and NSSI, suggesting distinct emotional pathways underlying these behaviors.

From a clinical and educational perspective, these results underscore the importance of early identification of emotional regulation difficulties in school settings and support the implementation of preventive, school-based interventions aimed at strengthening core socioemotional competencies. Early adolescence may represent a critical window for prevention, in which addressing emotional vulnerability—particularly among youth with elevated ADHD-related symptoms—could help reduce the risk of subsequent suicidal and self-injurious behaviors.

## Availability of Data and Materials

The datasets used and/or analyzed during the current study are available from the corresponding author on reasonable request.

## References

[1] Muela A, Bryan CJ, García-Ormaza J, Salaberria K (2024). Cross-cultural adaptation and psychometric validation of the Suicide Cognitions Scale-Revised (SCS-R) in Spanish adolescents in residential care. *The Spanish Journal of Psychology*.

[2] Balazs J, Kereszteny A (2017). Attention-deficit/hyperactivity disorder and suicide: A systematic review. *World Journal of Psychiatry*.

[3] Austgulen A, Posserud MB, Hysing M, Haavik J, Lundervold AJ (2024). Deliberate self-harm in adolescents screening positive for attention-deficit/hyperactivity disorder: A population-based study. *BMC Psychiatry*.

[4] Austgulen A, Skram NKG, Haavik J (2023). Risk factors of suicidal spectrum behaviors in adults and adolescents with attention-deficit/hyperactivity disorder: A systematic review. *BMC Psychiatry*.

[5] Impey M, Heun R (2012). Completed suicide, ideation and attempt in attention deficit hyperactivity disorder. *Acta Psychiatrica Scandinavica*.

[6] Septier M, Stordeur C, Zhang J, Delorme R, Cortese S (2019). Association between suicidal spectrum behaviors and Attention-Deficit/Hyperactivity Disorder: A systematic review and meta-analysis. *Neuroscience and Biobehavioral Reviews*.

[7] Balázs J, Gádoros J (2005). Comorbidity in child psychiatry: Is the comorbidity of pediatric mania and ADHD really that high?. *Psychiatria Hungarica : A Magyar Pszichiátriai Társaság Tudományos Folyóirata*.

[8] O’Connor RC, Kirtley OJ (2018). The integrated motivational–volitional model of suicidal behaviour. *Philosophical Transactions of the Royal Society of London. Series B, Biological Sciences*.

[9] Hawton K, Bale L, Brand F, Townsend E, Ness J, Waters K (2020). Mortality in children and adolescents following presentation to hospital after non-fatal self-harm in the Multicentre Study of Self-harm: A prospective observational cohort study. *The Lancet. Child & Adolescent Health*.

[10] Muela A, García-Ormaza J, Sansinenea E (2024). Suicidal behavior and deliberate self-harm: A major challenge for youth residential care in Spain. *Children and Youth Services Review*.

[11] Ribeiro JD, Franklin JC, Fox KR, Bentley KH, Kleiman EM, Chang BP (2016). Self-injurious thoughts and behaviors as risk factors for future suicide ideation, attempts, and death: A meta-analysis of longitudinal studies. *Psychological Medicine*.

[12] Winstone L, Heron J, John A, Kirtley O, Moran P, Muehlenkamp J (2024). Ecological momentary assessment of self-harm thoughts and behaviors: Systematic review of constructs from the Integrated Motivational-Volitional Model. *JMIR Mental Health*.

[13] Witt K, Milner A, Spittal MJ, Hetrick S, Robinson J, Pirkis J (2019). Population attributable risk of factors associated with the repetition of self-harm behaviour in young people presenting to clinical services: A systematic review and meta-analysis. *European Child & Adolescent Psychiatry*.

[14] Moran P, Chandler A, Dudgeon P, Kirtley OJ, Knipe D, Pirkis J (2024). The Lancet Commission on self-harm. *Lancet (London, England)*.

[15] Taliaferro LA, Almeida J, Aguinaldo LD, McManama O’Brien KH (2019). Function and progression of non-suicidal self-injury and relationship with suicide attempts: A qualitative investigation with an adolescent clinical sample. *Clinical Child Psychology and Psychiatry*.

[16] Allely CS (2014). The association of ADHD symptoms to self-harm behaviours: A systematic PRISMA review. *BMC Psychiatry*.

[17] Ward JH, Curran S (2021). Self-harm as the first presentation of attention deficit hyperactivity disorder in adolescents. *Child and Adolescent Mental Health*.

[18] Blasco-Fontecilla H, Ramos JM, López-Ibor MI, Chiclana-Actis C, Faraco M, González-Cabrera J (2025). Rising rate of hospitalizations in adolescents with Attention Deficit Hyperactive Disorder in Spain. *Journal of Attention Disorders*.

[19] Soriano V, Ramos JM, López-Ibor MI, Chiclana-Actis C, Faraco M, González-Cabrera J (2024). Trends in suicidal behavior among hospitalized adolescents in Spain over two decades. *Journal of Affective Disorders*.

[20] Soriano V, Ramos JM, López-Ibor MI, Chiclana-Actis C, Faraco M, González-Cabrera J (2025). Hospital admissions in adolescents with mental disorders in Spain over the last two decades: A mental health crisis?. *European Child & Adolescent Psychiatry*.

[21] Levy T, Kronenberg S, Crosbie J, Schachar RJ (2020). Attention-deficit/hyperactivity disorder (ADHD) symptoms and suicidality in children: The mediating role of depression, irritability and anxiety symptoms. *Journal of Affective Disorders*.

[22] Faraone SV, Rostain AL, Blader J, Busch B, Childress AC, Connor DF (2019). Practitioner Review: Emotional dysregulation in attention-deficit/hyperactivity disorder - Implications for clinical recognition and intervention. *Journal of Child Psychology and Psychiatry, and Allied Disciplines*.

[23] Bunford N, Evans SW, Wymbs F (2015). ADHD and emotion dysregulation among children and adolescents. *Clinical Child and Family Psychology Review*.

[24] Özbaran B, Kalyoncu T, Köse S (2018). Theory of mind and emotion regulation difficulties in children with ADHD. *Psychiatry Research*.

[25] Slaughter KE, Leaberry KD, Fogleman ND, Rosen PJ (2020). Reactive and proactive aggression in children with and without ADHD and negative emotional lability. *Social Development*.

[26] Fox KR, Harris JA, Wang SB, Millner AJ, Deming CA, Nock MK (2020). Self-Injurious Thoughts and Behaviors Interview-Revised: Development, reliability, and validity. *Psychological Assessment*.

[27] García-Ormaza J, Tavares J, Ammendola E, Muela A (2025). Mental pain and lifetime suicide attempts in early adolescence: A preliminary study. *Child and Adolescent Psychiatry and Mental Health*.

[28] García-Nieto R, Blasco-Fontecilla H, Yepes MP, Baca-García E (2013). Traducción y validación de la Self-Injurious Thoughts and Behaviors Interview en población española con conducta suicida. *Revista de Psiquiatría y Salud Mental*.

[29] Victor SE, Klonsky ED (2016). Validation of a brief version of the Difficulties in Emotion Regulation Scale (DERS-18) in five samples. *Journal of Psychopathology and Behavioral Assessment*.

[30] Gómez-Simón I, Penelo E, De La Osa N (2014). Factor structure and measurement invariance of the Difficulties in Emotion Regulation Scale (DERS) in Spanish adolescents. *Psicothema*.

[31] Sandín B (2003). Escalas PANAS de afecto positivo y negativo para niños y adolescentes (PANASN). *Revista de Psicopatología y Psicología Clínica*.

[32] Goodman R (1997). The Strengths and Difficulties Questionnaire: A research note. *Journal of Child Psychology and Psychiatry, and Allied Disciplines*.

[33] Ortuño-Sierra J, Chocarro E, Fonseca-Pedrero E, Sastre i Riba S, Muñiz J (2015). The assessment of emotional and behavioural problems: Internal structure of the Strengths and Difficulties Questionnaire. *International Journal of Clinical and Health Psychology : IJCHP*.

[34] Hall CL, Guo B, Valentine AZ, Groom MJ, Daley D, Sayal K (2019). The validity of the Strengths and Difficulties Questionnaire (SDQ) for children with ADHD symptoms. *PLoS One*.

[35] Biswas T, Scott JG, Munir K, Renzaho AMN, Rawal LB, Baxter J (2020). Global variation in the prevalence of suicidal ideation, anxiety and their correlates among adolescents: A population-based study of 82 countries. *EClinicalMedicine*.

[36] Nock MK, Green JG, Hwang I, McLaughlin KA, Sampson NA, Zaslavsky AM (2013). Prevalence, correlates, and treatment of lifetime suicidal behavior among adolescents: Results from the National Comorbidity Survey Replication Adolescent Supplement. *JAMA Psychiatry*.

[37] Wilkinson PO, Qiu T, Jesmont C, Neufeld SA, Kaur SP, Jones PB (2022). Age and gender effects on non-suicidal self-injury, and their interplay with psychological distress. *Journal of Affective Disorders*.

[38] Moloney F, Amini J, Sinyor M, Schaffer A, Lanctôt KL, Mitchell RHB (2024). Sex differences in the global prevalence of nonsuicidal self-injury in adolescents: A meta-analysis. *JAMA Network Open*.

[39] Gillies D, Christou MA, Dixon AC, Featherston OJ, Rapti I, Garcia-Anguita A (2018). Prevalence and characteristics of self-harm in adolescents: Meta-analyses of community-based studies 1990-2015. *Journal of the American Academy of Child and Adolescent Psychiatry*.

[40] Scott LN, Brown SL (2025). Revisiting the affect regulation model of suicide ideation: Bidirectional effects of momentary suicide ideation and affect. *Suicide & Life-threatening Behavior*.

[41] Coppersmith DDL, Millgram Y, Kleiman EM, Fortgang RG, Millner AJ, Frumkin MR (2023). Suicidal thinking as affect regulation. *Journal of Psychopathology and Clinical Science*.

[42] Wolff JC, Thompson E, Thomas SA, Nesi J, Bettis AH, Ransford B (2019). Emotion dysregulation and non-suicidal self-injury: A systematic review and meta-analysis. *European Psychiatry : the Journal of the Association of European Psychiatrists*.

[43] Paul E, Tsypes A, Eidlitz L, Ernhout C, Whitlock J (2015). Frequency and functions of non-suicidal self-injury: Associations with suicidal thoughts and behaviors. *Psychiatry Research*.

[44] Muela A, Balluerka N, Sansinenea E, Machimbarrena JM, García-Ormaza J, Ibarretxe N (2021). A social-emotional learning program for suicide prevention through animal-assisted intervention. Animals : An Open Access Journal from MDPI. *Animals : An Open Access Journal from MDPI*.

[45] Cicchetti D (1989). Developmental psychopathology: Some thoughts on its evolution. *Development and Psychopathology*.

[46] Cummings LR, Mattfeld AT, Pettit JW, McMakin DL (2021). Viewing nonsuicidal self-injury in adolescence through a developmental neuroscience lens: The impact of neural sensitivity to socioaffective pain and reward. *Clinical Psychological Science*.

[47] Kilford EJ, Garrett E, Blakemore SJ (2016). The development of social cognition in adolescence: An integrated perspective. *Neuroscience and Biobehavioral Reviews*.

[48] Dahl RE, Allen NB, Wilbrecht L, Suleiman AB (2018). Importance of investing in adolescence from a developmental science perspective. *Nature*.

[49] Steinberg L (2005). Cognitive and affective development in adolescence. *Trends in Cognitive Sciences*.

[50] Steinberg L (2008). A social neuroscience perspective on adolescent risk-taking. *Developmental Review : DR*.

[51] Crone EA, Dahl RE (2012). Understanding adolescence as a period of social–affective engagement and goal flexibility. *Nature Reviews. Neuroscience*.

[52] Forte A, Orri M, Galera C, Pompili M, Turecki G, Boivin M (2020). Developmental trajectories of childhood symptoms of hyperactivity/inattention and suicidal behavior during adolescence. *European Child & Adolescent Psychiatry*.

[53] Zanus C, Battistutta S, Aliverti R, Monasta L, Montico M, Ronfani L (2021). High-school students and self-injurious thoughts and behaviours: Clues of emotion dysregulation. *Italian Journal of Pediatrics*.

